# Comparing adult users of public and private dental services in the state of Minas Gerais, Brazil

**DOI:** 10.1186/1472-6831-14-100

**Published:** 2014-08-06

**Authors:** Rafaela da Silveira Pinto, Mauro Henrique Nogueira Guimarães de Abreu, Andrea Maria Duarte Vargas

**Affiliations:** 1 Universidade Federal de Minas Gerais, Avenida Antônio Carlos, CEP: 31270-901, 6627 Belo Horizonte, Brazil; 2 Secretaria de Estado de Saúde de Minas Gerais, Rodovia Pref., Américo Gianetti, CEP: 31630-900, 4143 Belo Horizonte, Brazil

**Keywords:** Use of dental services, Oral health, Adult, Brazil

## Abstract

**Background:**

Studying the factors associated with the use of dental services can provide the necessary knowledge to understand the reasons why individuals seek out public healthcare services and the formulation of more appropriate public policies for the present-day reality.

**Methods:**

This work was a cross-sectional epidemiological study consisting of a sample of adults found in a research databank concerning the conditions of the oral health of the population of the state of Minas Gerais, Brazil. This study examined both main oral health disorders and relevant socioeconomic aspects. The dependent variable was defined as the type of service used, categorized under public and private use. The independent variables were selected and grouped to be inserted in the analysis model according to an adaptation of the behavioral model described by Andersen and Davidson. A hierarchical model was used to analyze the data. The description of variables and bivariate analyses were performed in an attempt to verify possible associations. For each group of variables at each hierarchical level, the gross and adjusted odds ratios (OR) and the respective 95% confidence intervals (CI) were estimated by means of logistic regression. The Complex Samples model from the SPSS statistics program, version 19.0, was used to analyze the sample framework.

**Results:**

In the final model, the factors associated with the use of public healthcare services by adults were directly related to the socioeconomic and demographic conditions of the individuals, including: being of a dark-skinned black race/color, belonging to families with more than four household residents and with a lower income level, residing in small towns, having more teeth that need treatment.

**Conclusions:**

According to the findings from this study, socioeconomic and demographic factors, as well as normative treatment needs, are associated with the use of public dental services.

## Background

A wide range of theoretical models have attempted to explain the use of healthcare services by the general population, such as the Health Belief Model [1], the Dutton Model [2], the Evans and Stoddart Model [3], as well as the diverse stages of Andersen’s Behavioral Model [4-6]. The model proposed by Andersen and Newman [5] has been the most commonly applied model in both use and access studies. The greater applicability can be explained by its relatively easy implementation and its constant updating over recent decades. This model is considered to be the pioneer that has influenced all other models [7].

More recently, as regards the issues of oral health, an adaptation of the original behavioral model was developed [8]. This model proposes that the external environmental characteristics, the characteristics of the healthcare system, and the characteristics of the general population influence oral health and suggest the application of the healthcare management model to describe, predict, and explain the behavior of the population regarding healthcare and the health conditions of the population.

The Brazilian adult, ranging from 35 to 44 years of age, presents a DMFT of 16.75, with 7.1% never having gone to the dentist. Upon analyzing the type of service used, of those who consulted a dentist, 38.3% of the adults sought out public healthcare services [9]. In the state of Minas Gerais, the oral health conditions of adults are quite similar, with a DMFT of 15.9, with 4.5% never having gone to the dentist, and of those who had gone, 31.8% used public healthcare services [10].

Oral problems represent the third most prevalent reason for individuals to seek out healthcare services, though the inequality of services still exists [11].

Many researchers have dedicated themselves to studying the population’s use of healthcare services [12-21]. As regards Brazilian studies, the research has focused on the use of healthcare services in general [22-24] and in the elderly population [25-27], the regular use of dental services [25,28-30], the use of dental services due to pain [31], and the characterization of the use of dental services [32-34], without highlighting the type of service. Only two studies were found in the literature with outcomes similar to the dental services (public or private) used by the adult population, illustrating that there are still unanswered questions in this theme [16,35].

The phenomenon of the use of oral health services is a complex issue, especially in Brazil, where young age groups are given priority over adults and the elderly in public services [36].

With the creation of the Unified Health System, and later with the National Oral Health Policy, it has therefore become essential to analyze the characteristics associated with the type of service used by the adult population, given that oral healthcare services must be well-structured to attend to the existing demand. In this sense, studying the factors associated with the use of dental services can provide the knowledge necessary to understand the reasons why individuals seek out public services and the formulation of more appropriate public policies for the present-day reality.

Therefore, this work proposes to investigate the factors associated with the use of public dental services by the adult population of the state of Minas Gerais, Brazil, using data from the SB Minas Gerais Project – research on the oral health conditions of the population of Minas Gerais, based on Andersen and Davidson’s (1997) [8] theoretical model.

## Methods

### Studied area

The state of Minas Gerais is located in the southeastern region of Brazil. According to the 2012 population estimate carried out by the Brazilian Institute of Geography and Statistics, Minas Gerais has a population of 19,855,332 inhabitants [37] and is the second largest state in Brazil in number of inhabitants. In December 2012, the state had 14,252 dentists, which shows a ratio of one dentist for approximately every 1.394 inhabitants. Of this total number of registered dentists, 49.5% worked in the Brazilian Unified Health System [38]. As regards the coverage of health insurance plans, data from the National Supplementary Health Agency, responsible for regulating private health insurance plans, indicate that, in 2012, 26.3% of the residents in Minas Gerais had a health insurance plan, while only 7.1% had dental health insurance plans [39]. In the public sector, at the end of 2012, the state reported having 2,568 oral healthcare teams in the Family Health Strategy program, whose main activities are in primary healthcare, which represented population coverage of 38.8% [40]. When other dentists who work with primary care in the Brazilian Unified Health System were considered in the calculation of the population’s coverage, this number rose to 54.2% [41].

### Data source

This study used the databank from the SB Minas Gerais Project – research on the oral health conditions of the population of Minas Gerais [10], a cross-sectional study that investigated the main oral health disorders, as well as the relevant socioeconomic aspects, following the national methodology [9], for ages 5–12, as well as age groups of 15–19, 35–44, and 65–74. In an attempt to maintain the same methodological basis, the process used was the same as the national survey [9], considering that the sample size was also based on the severity of dental caries, as estimated by DMFT, but in this case, relied only on the data from the SBBrasil 2010 for the southeastern region of Brazil. For each age group and each domain, the prevalence of caries and the DMFT average were used as a reference for the calculation of the sample size associated with a set margin of error. The proposed design ensures the formulation of inferences to estimate the number of dental caries for the state of Minas Gerais and for each domain, considering each age or age group. For other healthcare problems, the degree of representativeness will vary according to the estimated prevalence and severity. The overall response rate was of 81.1%, thus falling within the parameters established within the sample plan. The framework of the sample plan referent to the databank is available in the project’s final report [10].

The inquiry included a representative sample from the state of Minas Gerais. The *a posteriori* sample calculation showed that the researched sample ensures a 95% confidence interval (CI), with a power of 80%.

As the present study’s dependent variable proved to be different from that used in the sample calculation from the initial project, many subsequent calculations were performed *a posteriori*, using data from adults surveyed in the SB Brasil 2010 [9] (databank on which the sample calculation of the SB Minas Gerais [10] was based). Some examples included the expected frequency of the income variable, which was 20.6% in the public sector and 79.4% in the private sector; the education level variable, whose frequency reached 29% in the public sector and 71% in the private sector; and the pain variable, whose expected frequency reached 51.7% and 48.3%, respectively. Comparing the variables of income, education level, and pain among users from the public and private sectors, considering an existing sample of 8,978 adults, a minimum power of 80% (20% of type II errors) and the 95% significance level (5% of type I error) were ensured.

### Study variables

The theoretical model set forth by Andersen and Davidson (1997) [8] is an adaptation of the behavioral model created by Andersen and Newman (1973) [5] for oral health studies. Its precursor proposes that individual determining factors of the use of services are divided into three categories: predisposing, enabling, and self-reported level of the disease.

The predisposing factors are associated with the tendency of the individual to use the services and can be:

– *Demographic:* age, sex, marital status, prior history of the disease. People with different ages present different demands, and those that have a history of the disease generally require a more frequent use of the services.

– *Social Structure:* education level, race, occupation, family size, ethnicity, religion, residential mobility. Data such as education and occupation of the head of the family can reflect the lifestyle of the individuals.

– *Beliefs:* values relevant to the health/disease process, knowledge of the disease, attitudes, considering that the individual perception concerning these questions influences an individual’s behavior.

The capacitating factors are those that make the resources from healthcare services available to the individual and can be relevant to both the family and the community:

– *Family:* income, degree of coverage of healthcare insurance plans, an individual’s access to regular care and to the nature of this regular care.

– *Community:* taxes levied on healthcare services, the price of services, the region of the country, urban/rural characteristics.

The level of the disease is associated with the perception of the disease and its possibility to occur. This can be self-referred (lack of appetite, symptoms, general state) or evaluated by the professional (symptoms, diagnosis).

As regards the use of healthcare services, which is the result of this model, the type, the purpose, and the analysis unit must be analyzed. This type of service refers to the hospital, the doctor, the dental surgeon, the pharmacy, doctor’s home visits, among others. This proposal is related to the reason for the patient to search for healthcare service, be it for prevention (primary care), to promote functionality (secondary care), and to stabilize irreversible diseases (tertiary care). The analysis unit, however, refers to the degree of contact, the volume, and whether or not it is temporary care.

The participants of the SB Minas Gerais Project [10] were asked if they had ever been to the dentist in their life and, if the answer was ‘yes’, what type of service was used during the last appointment. Those who answered ‘yes’ to the first question were considered eligible to participate in this study.

The dependent variable was the type of service used, which could be characterized as public, private, healthcare plan, and others. Those who answered “others” were excluded from the study, since the nature of this type of classification is unknown. For this reason, the original variable was redefined under the categories of Public Healthcare Services and Private Healthcare Services (which includes services rendered for private healthcare and health insurance plans).

The independent variables were selected and grouped to be inserted in the model of analysis according to an adaptation of the behavioral model formulated by Andersen and Davidson [8].

The group of variables referent to predisposing factors (most distal factors) included gender, age (35–39 and 40–44 years of age), education level (none, 1–4, 5–8, 9–11, and 12–15 complete years of study), race/color (whites, dark-skinned blacks, other – which included Asian descendants, light-skinned blacks, and Amerindians), and the number of household residents (from 1 to 4 people and more than 4).

The group of variables referent to the enabling factors included family monthly income, converted into dollars, considering US$1.00 = R$2.00 [42] (≤$750.00; $751.00 to $1,250.00; ≥$1,251.00/month). Also considered was the size of the town in number of inhabitants (≤10,000; 10,001-40,000; ≥40,001; and the state capital).

Regarding the variable referent to the reported level of disease, this study considered the self-assessment of oral health (very satisfied/satisfied, not satisfied/not unsatisfied, unsatisfied/very unsatisfied), self-reported need for dental treatment (yes or no), complaint of toothache within the past 6 months (yes or no), and the self-reported need for a total prosthesis (yes or no).

As regards the variables of the need for dental treatment evaluated by a healthcare professional, this study used the need for prosthesis (no need, need for one or more partial prostheses, need for one or more total prostheses, need for both partial and total prostheses), the number of teeth needing treatment, the presence of periodontal problems (lesser complexity – when the individual possesses one of the following conditions as the highest CPI index: healthy, bleeding, tartar, not examined, excluded sixth; higher complexity – when the individual presented the highest CPI index for periodontal pockets of 4–5 mm or more than 6 mm).

The following variables were considered regarding the characterization of the service (proximal factors): reason for the patient’s last dentist appointment (check up/prevention or oral problems – pain, extraction, treatment, others) and time elapsed since the patient’s last dentist appointment (less than one year or more than one year).

### Data analysis

To analyze the data, this study used the model proposed by Victora et al. (1997), which considers that there are proximal or distal factors associated with the outcome, considering that distal factors influence proximal factors, measuring their effect and controlling possible confounding factors [43].

All statistical analyses were developed in the Complex Samples module of the SPSS program, version 19.0 [44], considering the sample framework used in this study. The description of the variables was performed in the first stage, while Pearson’s chi-squared test, with corrections implemented by Rao-Scott, and the Student’s t test for independent samples were used to verify the existence of possible associations.

**Figure 1:**
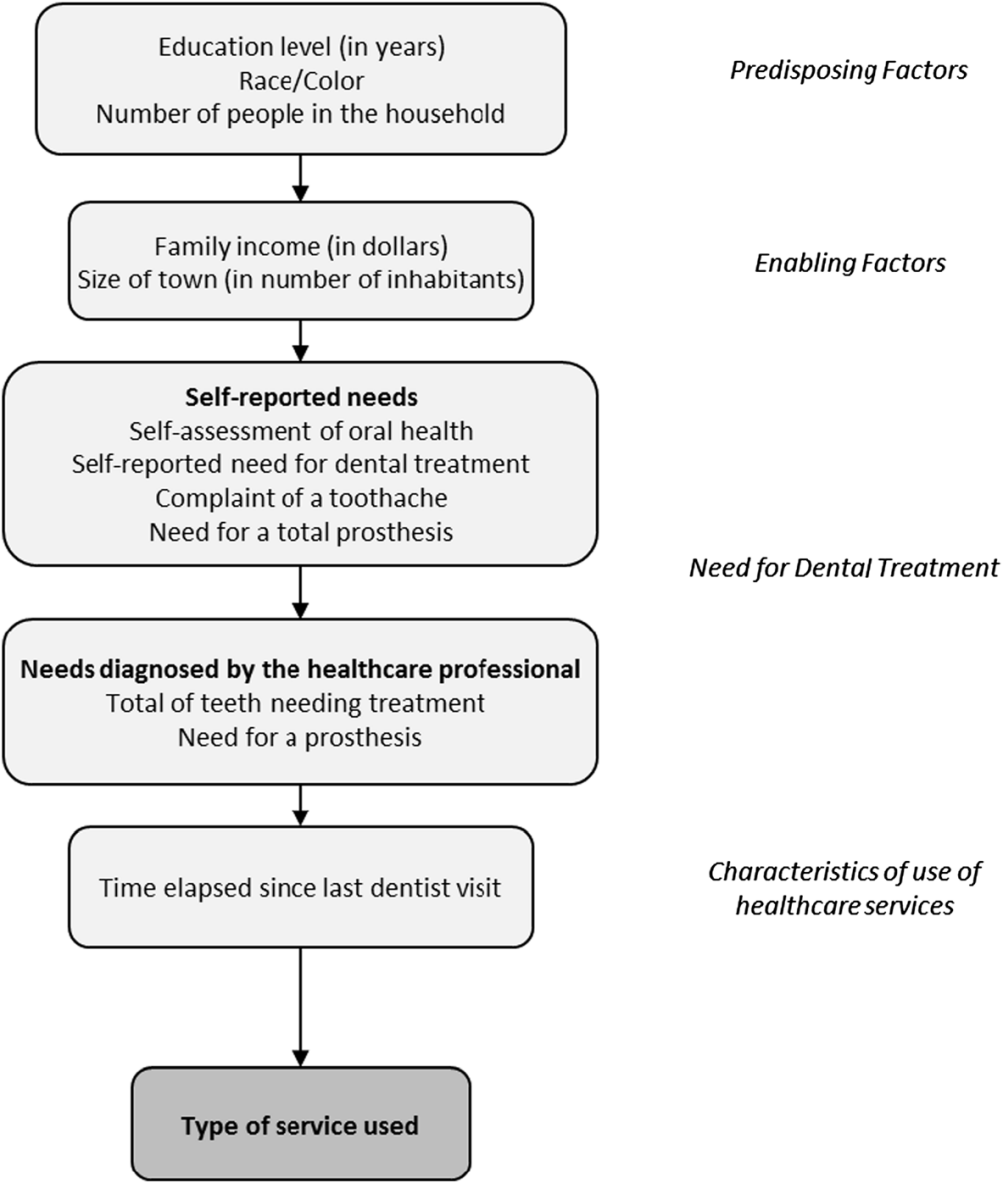
Model of analysis.

According to Victora et al. (1997) [43], this approach includes a first model with the more distal level variables. At this level, the co-variables that are associated with the outcome of the level p < 0.20 can be included at the next level, and so forth, until reaching the more proximal co-variable level. In the present study’s case, the order of insertion of the variables in the model included: predisposing factors, enabling factors, self-reported level of disease, level of disease evaluated by the healthcare professional, and the characterization of the healthcare service (Figure 1). Model 1 included ‘Education level (in years)’, ‘Race/Color’, ‘Number of people in the household’ – predisposing factors. Their measures of effect were assessed in this first model. Those variables that reached p value <0.20 were kept in model 2. In model 2, ‘Family income (in dollars)’ and ‘Size of town (in number of inhabitants)’ – enabling factors were included together with those kept in model 1. Those variables that reached p value <0.20 were kept in the model 3. In model 3, ‘self-assessment of oral health’, ‘Self-reported need for dental treatment’, ‘Complaint of a toothache’, ‘Need for a total prosthesis’ - Self-reported needs were included together with those variables kept in model 2. Those variables that reached p value <0.20 were kept in the model 4. In Model 4, ‘Need for a prosthesis’ and ‘Total of teeth needing treatment’ - Needs diagnosed by the healthcare professional were included together with those kept in model 3. Those variables that reached p value <0.20 were kept in the model 5. In model 5, ‘Time elapsed since last dentist visit’ - Characteristics of use of healthcare services were included together with those kept in model 4. According to recommendations formulated by Hosmer & Lemeshow (2005) [45], only variables with a p < 0.05 were maintained in the final model, since the maintenance of other co-variables in the final model would change the estimates.

### Ethical implications

This study was approved by the Research Ethics Committee from the Federal University of Minas Gerais (UFMG), under protocol number 501.115/2013.

## Results

This work selected 1,207 adults to participate in this study. Of these, 1,102 reported having received public or private healthcare/health insurance services, while the remainder reported having used “other” types of services or never having had a dentist appointment, and were therefore excluded from this study, as they did not fit the scope of this analysis.

The participants of this study were mostly female (65.8%), at an average of 39.3 years of age (SD = 0.1 years); 19.3% reported an educational level of up to 4th grade (0.8% never went to school), with an average of 9 years of study (SD-0.2); 45% of the participants were white; 56.3% were individuals with a family income of up to $750,00; and the average number of household residents was up to 4 people (68.0%). On average, 1.72 (SD = 0.13) teeth needed treatment.

Table 1 describes the predisposing and enabling factors, as well as health needs and characterization of the healthcare services according to the type of service used. The variables of gender, age, presence of periodontal problem, and reason for dentist appointment were not associated with the type of service used.

**Table 1:** Distribution of user of dental services according to predisposing and enabling factors, need for dental treatment, and characteristics of use of healthcare services, SB Minas Gerais Project, 2012

**Variables**	**Use of public health care services**		**Use of private healthcare services**		**p value**
	**n**	**%**	**n**	**%**	
*Predisposing factors*					
**Gender**					
Female	264	67.3	460	65.1	**0.594**
Male	121	32.7	256	34.9	
**Age**					
40-44	170	47.3	351	47.4	**0.978**
35-39	215	52.7	365	52.6	
**Education level (in years)**					
12-15	74	20.3	223	33.8	**<0.001**
9-11	79	21.0	200	27.8	
5-8	109	32.4	177	22.5	
1-4	112	24.9	105	15.4	
None	10	1.5	9	0.5	
**Race/color**					
Whites	131	37.6	316	48.5	**0.001**
Dark-skinned blacks	60	16.2	58	7.2	
Others	194	46.2	342	44.3	
**Number of household residents**					
From 1 to 4	225	56.4	518	73.5	**<0.001**
More than 4	160	43.6	198	26.5	
*Enabling factors*					
**Family income (in dollars)**					
More than $1,251.00	19	7.9	147	23.9	**<0.001**
from $751,00 to $1,250.00	68	23.4	184	29.7	
Up to $750,00	294	68.7	374	46.5	
**Size of town (in number of inhabitants)**					
State Capital	60	9.4	192	14.4	**0.105**
> 40 thousand	124	57.9	270	60.7	
10-40 thousand	107	22.1	151	18,1	
<10 thousand	94	10.6	103	6.9	
*Need for dental treatment*					
**Self-reported needs**					
**Self-assessment of oral health**					
Unsatisfied/very unsatisfied	118	33.2	216	29.2	**0.166**
Not satisfied/not unsatisfied	77	20.8	122	17.3	
Satisfied/very satisfied	186	46.0	376	53.5	
**Self-reported need for dental treatment**					
No	88	24.9	216	31.6	**0.047**
Yes	290	75.1	487	68.4	
**Complaint of a toothache**					
No	261	71.3	577	81.7	**0.002**
Yes	123	28.7	137	18.3	
**Need for a total prosthesis**					
No	239	67.0	518	72.5	**0.176**
Yes	138	33.0	181	27.5	
**Needs diagnosed by the healthcare professional**					
**Need for a prosthesis**					
No need	121	34.4	327	45.2	**0.113**
One or more partial prostheses	237	62.1	355	52.7	
One or more total prostheses	8	2.0	13	1.3	
Partial and total prostheses	10	1.6	10	0.9	
**Presence of periodontal problem**					
Higher complexity	59	16.6	104	15.4	**0.693**
Lower complexity	326	83.4	612	84.6	
**Teeth needing treatment (Mean, SD)**	2,44	0.237	1.37	1.127	**<0.001**
*Characteristics of use of healthcare services*					
**Reason for dentist appointment**					
Check up/prevention	156	22.6	75	21.0	**0.629**
Oral problems	560	77.4	310	79.0	
**Time elapsed since last dentist appointment**					
Less than 1 year	174	40.2	350	51.8	**0.006**
More than 1 year	209	59.8	361	48.2	

Table 2 shows the various stages of the insertion of the variables, according to the proposed hierarchical model. In the first column, one can verify the gross OR, obtained by means of a bivariate analysis of the outcome. Further down, one can see the analysis of the predisposing factors. The variables that maintained the value of p < 0.20 at this level of analysis were thereby included in the next level, which was continued successively until reaching the level of the variables that describe the characterization of the services.

**Table 2:** Results from the multivariate analysis for groups of predisposing and enabling factors, need for dental treatment, and characteristics of use of dental services, SB Minas Gerais Project, 2012

**Variable**	**Unadsjusted OR (95% CI) p value**	**Adjusted OR**^**1 **^**(95% CI) p value**	**Adjusted OR**^**2 **^**(95% CI) p value**	**Adjusted OR**^**3 **^**(95% CI) p value**	**Adjusted OR**^**4 **^**(95% CI) p value**	**Adjusted OR**^**5 **^**(95% CI) p value**
		**Model 1**	**Model 2**	**Model 3**	**Model 4**	**Model 5**
*Predisposing Factors*						
**Education level (in years)**						
12-15	1.0	1.0	1.0	1.0	1.0	1.0
9-11	1.26 (0.79-2.02) 0.338	1.23 (0.78-1.94) 0.360	1.04 (0.66-1.64) 0.854	1.06 (0.66-1.69) 0.823	1.01 (0.64-1.61) 0.956	1.01 (0.65-1.59) 0.958
5-8	2.39 (1.47-3.89) 0.001	1.99 (1.23-3.22) 0.005	1.51 (0.90-2.52) 0.115	1.51 (0.90-2.53) 0.121	1.36 (0.82-2.26) 0.227	1.42 (0.85-2.39) 0.183
1-4	2.68 (1.58-4.55) <0.001	2.43 (1.46-4.06) 0.001	1.64 (0.96-2.83) 0.072	1.62 (0.92-2.83) 0.092	1.47 (0.86-2.50) 0.159	1.50 (0.87-2.61) 0.143
none	5.23 (1.59-17.16) 0.007	3.51 (1.01-12.15) 0.047	2.11 (0.60-7.41) 0.240	2.15 (0.58-7.95) 0.251	1.83 (0.52-6.44) 0.341	1.57 (0.47-5.22) 0.463
**Race/Color**						
Whites	1.0	1.0	1.0	1.0	1.0	1.0
Dark-skinned blacks	2.93 (1.71-5.01) <0.001	2.42 (1.41-4.14) 0.001	2.40 (1.35-4.26) 0.003	2.31 (1.27-4.20) 0.006	2.28 (1.26-4.10) 0.007	2.44 (1.35-4.41) 0.003
Others	1.35 (0.92-1.97) 0.125	1.23 (0.83-1.84) 0.301	1.18 (0.78-1.79) 0.426	1.11 (0.71-1.72) 0.646	1.09 (0.73-1.62) 0.683	1.10 (0.73-1.65) 0.638
**Number of people in the household**						
From 1 to 4	1.0	1.0	1.0	1.0	1.0	1.0
More than 4	2.14 (1.53-3.01) <0.001	1.84 (1.27-2.65) 0.001	1.95 (1.35-4.26) 0.001	1.84 (1.24-2.72) 0.003	1.92 (1.32-2.78)0.001	1.91 (1.32-2.76) 0.001
*Enabling Factors*						
**Family income (in dollars)**						
More than $1,251.00	1.0		1.0	1.0	1.0	1.0
From $751.00 to $1,250.00	2.38 (1.07-5.30) 0.034		2.21 (1.04-4.71) 0.040	2.25 (1.04-4.83) 0.039	2.08 (1.03-4.19) 0.040	2.16 (1.00-4.66) 0.050
Up to $750.00	4.46 (2.04-9.72) <0.001		3.65 (1.68-7.94) 0.001	3.61 (1.65-7.92) 0.002	3.22 (1.57-6.56) 0.002	3.25 (1.44-7.35) 0.005
**Size of town (in number of inhabitants)**						
State Capital	1.0		1.0	1.0	1.0	1.0
> 40 thousand	1.46 (0.93-2.29) 0.098		1.86 (1.17-2.94) 0.009	1.94 (1.21-3.12) 0.007	1.80 (1.13-2.87) 0.014	1.76 (1.11-2.79) 0.016
10-40 thousand	1.87 (1.06-3.28) 0.030		1.95 (1.11-3.46) 0.022	1.97 (1.09-3.54) 0.025	1.90 (1.04-3.46) 0.036	1.97 (1.10-3.51) 0.022
<10 thousand	2.37 (1.41-3.97) 0.001		2.72 (1.65-4.49) <0.001	2.74 (1.63-4.60) <0.001	2.96 (1.77-4.95) <0.001	2.86 (1.72-4.74) <0.001
*Need for Dental Treatment*						
**Self-reported needs**						
**Self-assessment of oral health**						
Unsatisfied/Very Unsatisfied	1.0			1.0	-	-
Not satisfied/Not Unsatisfied	1.06 (0.65-1.73) 0.810			1.22 (0.73-2.06) 0.445	-	-
Satisfied/Very Satitisfied	0.76 (0.52-1.10) 0.144			1.00 (0.62-1.60) 0.997	-	-
**Self-reported need for dental treatment**						
No	1.0			1.0	-	-
Yes	1.39 (1.00-1.93) 0.048			0,90 (0.60-1.37) 0.629	-	-
**Complaint of a toothache**						
No	1.0			1.0	1.0	-
Yes	1.80 (1.24-2.61) 0.002			1.47 (0.95-2.28) 0.080	1.28 (0.81-2.01) 0.286	-
**Need for a total prosthesis**						
No	1.0			1.0	-	-
Yes	1.30 (0.89-1.89) 0.177			0.91 (0.59-1.38) 0.640	-	-
**Needs diagnosed by the healthcare professional**						
**Need for a prosthesis**						
No need	1.0				1.0	-
One or more partial prostheses	1.55 (1.01-2.39) 0.045				1.03 (0.64-1.67) 0.895	-
One or more total prostheses	2.08 (0.44-9.82) 0.351				1.97 (0.31-12,46) 0.470	-
Partial and total prostheses	2.32 (0.65-8.29) 0.192				0,89 (0.21-3.71) 0.871	-
**Total of teeth needing treatment**	1.13 (1.06 – 1.20) <0.001				1.08 (1.00-1.16) 0.052	1.08 (1.01-1.15) 0.030
*Characteristics of use of healthcare services*						
**Time elapsed since last dentist visit**						
Less than 1 year	1.0					1.0
More than 1 year	1.60 (1.15-2.23) 0.006					1.13 (0.81-1.58) 0.464

^1^Adjustment among predisposing factors ^2^Enabling factors, adjusted among themselves and by the predisposing factors ^3^Self-reported need for dental treatment, adjusted among themselves and by the predisposing and enabling factors ^4^Need for dental treatment (diagnosed by the professional), adjusted among themselves and by the predisposing and enabling factors as well as by the self-reported need for dental treatment ^5^Characteristics of the use of dental services, adjusted among themselves and by the predisposing and enabling factors as well as the need for dental treatment (self-reported and diagnosed by the healthcare professional).

Regarding the predisposing factors, it is possible to perceive that all of the variables remain significant up to the final level of analysis. The same is true for the enabling factors. When the self-reported needs are inserted into the model, only the complaint of a toothache remained statistically significant, given that the chance of someone with a toothache using public healthcare services was 32% more likely than for those who did not complain of a toothache. Nevertheless, this single variable remained statistically significant only at the next level of analysis when the diagnostic needs reported by the healthcare professional were inserted. These last variables, not including the number of teeth needing treatment, also were not considered statistically significant. The average number of teeth needing treatment was of 1.68, given that when a tooth needing treatment was added to the calculation, the chance of using public healthcare services increased by 8%. When the variable of time elapsed since the last dentist appointment was inserted, representing the characteristics of the healthcare services, this variable diminished in statistical significance.

Table 3 illustrates that, in the final model, the factors associated with the use of public healthcare services by adults are preponderantly those related to the socioeconomic and demographic factors of the individuals. In this sense, people of dark-skinned black race/color are approximately 2.5 times more likely to use public healthcare services than are individuals who declare themselves to be white. As regards the number of household residents, larger families (with more than 4 residents) are approximately twice as likely to use public healthcare services. As regards family income, the individuals with a higher family income (up to $750,00) are 4 times more likely to use public healthcare services. The variable of “size of town” shows a result similar to that of family income, given that the inhabitants of small towns proved to be 3 times more likely to use public healthcare services than were inhabitants of the state capital. Concerning the number of teeth needing treatment, in the final model, it could be observed that with each increment of 1 tooth needing treatment, the chance of using public healthcare services increased by 9%.

**Table 3:** Final result of the multivariate analysis for groups of predisposing and enabling factors, need for dental treatment, and characteristics of use of dental services

**Variables**	**OR (95% CI)**	**p value**
*Predisposing factors*		
**Race/Color**		
Whites	1.0	
Dark-skinned blacks	2.41 (1.29-4.50)	0.006
Others	1.12 (0.75-1.66)	0.580
**Number of people in the household**		
From 1 to 4	1.0	
More than 4	1.98 (1.38-2.85)	<0.001
*Enabling Factors*		
**Family income (in dollars)**		
More than $1,251.00	1.0	
From $751.00 to $1,250.00	2.28 (1.05-4.96)	0.039
Up to $750.00	3.87 (1.77-8.46)	0.001
**Size of town (in number of inhabitants)**		
State Capital	1.0	
> 40 thousand	1.73 (1.10-2.71)	0.018
10-40 thousand	1.96 (1.11-3.44)	0.020
<10 thousand	2.95 (1.89-4.64)	<0.001
*Need for Dental Treatment*		
**Total of teeth needing treatment**	1.09 (1.02-1.17)	0.009

SB Minas Gerais Project, 2012.

## Discussion

In the present study, the greatest chance of using public healthcare services was related to the dark-skinned black race/color, the greater number of household residents, a lower family income, inhabitants of small towns, and having a larger number of teeth needing treatment.

Evidence shows that there may be differences in the quantity and nature of service providers when these are classified by race/color groups [46]. These racial inequalities have also been previously identified in oral healthcare services. The use of public healthcare services was different among dark-skinned blacks (56.8%), light-skinned blacks (53.3%), and whites (41.6%) [47], as was also verified in the present study. In the United States, results have also shown that the dark-skinned black population more commonly seeks out public healthcare services, while the white population more often uses private healthcare services, data which has remained unchanged over time [48,49]. The determining social factors can explain part of the worse access to and lesser use of oral healthcare services by the dark-skinned black population in general. The race may be considered a limiting factor for the use of oral healthcare services for the elderly as well, given that for dark-skinned black elderly people, the chance of never having gone to the dentist is more than twice that for the white population, and even if needing a prosthesis or if presenting pain, the difficulty in using public healthcare services does not diminish [50]. The Brazilian Unified Health System, in line with governmental initiatives to reduce social exclusion, has been playing its role to care for those who most need public healthcare services, thus justifying the principle of equality. Historically and most often poor, dark-skinned black populations [51] in the Brazilian reality tend to use public healthcare services more often.

The larger number of household residents increases the chances of using public healthcare services. This may well be explained by the fact that the greater the number of household residents, the greater the number of dependents within a single family income and, consequently, less possibility of paying for healthcare services. The decision of which type of service to use is many times based on the needs of each individual within the family and who depend on the family income and not only on the needs of the head of the family or individual breadwinner. In a study on the elderly’s use of general healthcare services in the Southern regions of Brazil, it was noted that elderly people who are a part of larger families use private healthcare services less. Moreover, the increase of one member in this family further diminishes the chances of these elderly people using this private network by 15% [52]. In Norway, larger families also showed a lesser use of healthcare services in general [53]. The number of children can influence this use, considering the assumption that the more children, the larger the family. Both positive and negative associations with the use of healthcare services, regardless of the type of service used, have been reported in the literature. The findings, however, have not been consistent, given that the data used to associate the variables may well be influenced by the current healthcare system [46].

Public policies concerning healthcare must consider the insertion of individuals within families, given that, if on one hand the interaction among the residents of a household represents social support, on the other, it points toward restrictions in the use of financial resources geared toward healthcare services when this number of residents rises [52].

The financial cost of dental services has been consistently maintained as a barrier for the use of oral healthcare services worldwide. The questions concerning the payment of treatments and of its connection with income are present in many countries, both developed and developing, and in distinct healthcare systems [19]. Different healthcare system frameworks reveal the complexity of these systems. The different explanatory factors can in fact show this diverse formatting of healthcare service networks [46].

Nonetheless, even in different healthcare systems around the world, the key underlying factor for the use of oral healthcare services has depended upon each individual’s income level [14,16,18-21,54,55]. In Brazil, even with its universal healthcare system [56], the same barriers relevant to individual income and, consequently, to the payment of healthcare services can be found [25-30,35,47,52,57,58]. The present study, therefore, identified a dose–response gradient for this variable, which reinforces its determination in the use of public healthcare services.

Regarding the size of the town of residence, the fact that smaller towns have less trouble implementing the Family Health Strategy [59] makes it so that these same towns possess a more encompassing coverage provided by oral healthcare teams for the population as a whole, and, consequently, better access to healthcare services [60,61]. Another determining factor may well be the fact that the population in smaller towns has less purchasing power, which can limit their search for private healthcare services [35].

People with greater normative treatment needs, which may lead to some form of symptomology, can perceive a worsening of one’s own oral health, in turn forcing them to search for dental healthcare services [62,63].

In the present study, when the number of teeth needing treatment was considered, the result was the same as the previously reported data: people with a lower purchasing power present greater dental treatment needs, and, judging by their existing socioeconomic conditions, search for public dental services.

Women tend to use these services more often, both for one’s general health [46] and for dental services [28-30,35]. Healthcare needs to play a determining role in the use of public healthcare services. In this sense, as women generally evaluate their state of health as poor, the greater use of public healthcare services on the part of women could be explained merely by this self-perceived need. However, in this study, when questioned about the type of service used, no association with gender could be identified. This may well be explained by the fact that, for women, the relationship between the socioeconomic level, their social integration, and their comprehension of the processes that can influence healthcare questions have yet to be fully clarified [64].

Regarding age, studies show that the older a person is, the more they tend to use healthcare services in general [46]. Concerning dental services, when regular use was analyzed, the relation between age and use inverted, with the services being used more often by young people [28,29]. In the present study, the non-existence of this association can be explained by the fact that this study examined age groups with very close ages, choosing not to distinguish among these differences.

As regards periodontal disease, the fact that it appeared when associated with the type of service used can be explained by the fact that periodontal disease, in most cases, is quite slow. Thus, it may not represent the perception of the problem and, consequently, may not drive one to seek out some form of healthcare service. Some authors suggest that the most reliable symptom to measure periodontal disease and that calls the attention of the user is the mobility of teeth [55,65].

During the analysis, according to the Andersen and Davidson (1997) model, other tested variables lost their association with the outcome.

Although consistent with the theoretical model used [8], the present study’s findings do not ensure the causal relations specified by the model, given that the databank that gave rise to the analysis resulted from a cross-sectional study. In this manner, the present study can demonstrate association but not the correct temporal sequence necessary to draw conclusions about the causal mechanisms.

Another limitation of this study is the fact that the dependent variable considers only the type of service used in the last consultation, which may well be an exception to the type of routinely used service. However, considering the random method of selecting individuals, it can be inferred that these situations present a random distribution around the average values [46].

This study’s results show that the type of service sought for dental services is associated with the socioeconomic conditions of the adults.

When the use of services is studied through the classification of different socioeconomic factors, the equity of the healthcare system in question can be indirectly assessed. In countries with systems that encompass policies for the universality of healthcare services, such as England, Canada, and Brazil, equity can be confirmed by the lower income population’s greater use of healthcare services [46].

Brazil has been gradually reducing inequality in the access to healthcare services. Hospitalization, which tends to be the most costly and most urgent of services that an individual may have to face, when done through the Brazilian Unified Health System, shows that the majority of hospital beds are occupied by the lower income population. Nevertheless, other services, such as doctor’s and dentist’s appointments are still predominantly found in private healthcare services [58]. In Minas Gerais, this result is no different. Of the adults who had had a dentist appointment at least once in their life, only 31.8% went to their most recent appointment at a public healthcare clinic [10].

The right to health is understood as a basic need, and guaranteeing this right must be the core aim of universal and equalitarian public policies, such as the Brazilian Unified Health System. This agency, upon taking on this fight, assumes the responsibility to develop proposals denominated as *positive discrimination*, where individuals, according to their own needs, including an array of actions to reduce or compensate inequality within a more all-encompassing policy, attempt to more effectively benefit the more vulnerable social groups. In this manner, in its plan of healthcare actions and activities, the Brazilian Unified Health System provides a means through which to compensate for the social inequalities generated by the social structure [66].

In the present study, upon verifying that it is the individuals with lower socioeconomic and healthcare conditions that most frequently use healthcare services, it could be concluded that what occurs within this agency is in fact a positive discrimination in favor of the most vulnerable social groups.

It is important to emphasize the role of the Brazilian Unified Health System in reducing the inequalities in healthcare and in providing universal access to healthcare; however, the limitations of the system as regards the resolution of problems from the population must also be observed. The oral healthcare services network must be held accountable for the healthcare needs of the general population, and for this, it must create an entrance door for a more effective primary care, access facilitated by geographically well-distributed services, and well-established mechanisms to regulate the flow of healthcare services in order to achieve the full integrity of the services rendered [67].

## Conclusions

According to the findings from the present study, an association could be found among the use of public healthcare services, socioeconomic conditions (predisposing factors of race/skin color and the number of residents per household; enabling factors of income and size of city), and unfavorable clinical treatment (number of teeth needing treatment), thus indicating that the Brazilian Unified Health System has been playing its role in its attempt to promote equality.

## Abbreviations

OR: Odds ratio; CI: Confidence interval; DMFT: Decayed, missing, and filled teeth; CPI: community periodontal index; USD: American dollar.

## Competing interests

The authors hereby declare that there are no conflicts of interest.

## Authors’ contributions

RSP contributed in the framework of the study, literature review, analysis, and interpretation of data. MHNGA contributed in the framework of this study, analysis, and interpretation of data. AMDV contributed in the framework of this study, analysis, and interpretation of data. All authors participated in the writing up of this text and have read and approved the final version of the manuscript.

## Pre-publication history

The pre-publication history for this paper can be accessed here:

http://www.biomedcentral.com/1472-6831/14/100/prepub
